# Complex-frequency waves: beat loss and win sensitivity

**DOI:** 10.1038/s41377-024-01388-3

**Published:** 2024-02-01

**Authors:** Qingqing Cheng, Tao Li

**Affiliations:** 1 School of Optical-Electrical and Computer Engineering, University of Shanghai for Science and Technology, Shanghai, 200093 China; 2 Department of Respiratory Diseases and Critical Medicine, Quzhou Hospital Affiliated to Wenzhou Medical University, Quzhou, 324000 China; 3 National Laboratory of Solid State Microstructures, College of Engineering and Applied Sciences, Nanjing University, Nanjing, 210093 China

**Keywords:** Nanophotonics and plasmonics, Imaging and sensing, Metamaterials

## Abstract

Recent experiments have demonstrated that synthesized complex-frequency waves can impart a virtual gain to molecule sensing systems, which can effectively restore information lost due to intrinsic molecular damping. The enhancement notably amplifies the signal of trace molecular vibrational fingerprints, thereby substantially improving the upper limit of sensitivity.

Spectral analysis has emerged as a powerful, non-invasive tool for molecular identification, gaining prominence in applications like COVID-19 detection^1^ due to its specificity and cost-effectiveness. Despite these advances, detecting trace molecules presents a substantial challenge. Traditional methods, such as surface plasmon resonance (SPR)^2^, a quasi-bound state in the continuum (QBIC)^3^, and FANO resonance^4^, have made strides in enhancing light-matter interactions to improve the sensitivity and quality of sensing systems^5^. However, the intrinsic molecular damping always weakens these interactions, posing a significant hurdle in trace molecule detection. To overcome this, additional optical gain materials are being explored as a means to compensate for the damping^6^, aiming to overcome the limitation in sensing technologies. While the approach shows promise, it also introduces challenges such as increased interference and instability^7^, which are unfavourable to the detection process.

Complex-frequency waves (CFW) with temporally attenuation characteristics can impart virtual gain to systems, effectively mitigating the information that is lost due to intrinsic system losses^8^. In fact, a geneous design of superlens composed of negative index material with metallic inclusions has been hindered towards wide applications for a couple of decades due to large metal loss^9,10^. The CFW is quite promising to compensate for the loss and empower the superlens applications, nevertheless, there are still challenges in experimentally realizing CFW in the time domain. In a recent groundbreaking development^11^, Guan et al. have successfully addressed these issues by synthesizing truncated CFW across multiple frequencies. Their method involves treating the CFW as a coherent amalgamation of several real frequency waves. By measuring the optical response at various real frequencies and adhering to the Lorentzian lineshape, they recombine responses from different frequencies. The process culminates in the numerical synthesis of the optical response under complex frequency excitation. The innovative multi-frequency synthetic approach to truncated CFW introduces virtual gains into superlens imaging^11^ and surface plasmon polaritons propagation^12^, effectively overcoming the metal or plasmonic losses of systems.

As for trace molecule detection, the intrinsic damping loss in molecular materials significantly diminishes the interaction between molecular vibrational modes and plasmons. Specifically, the intrinsic damping broadens the vibrational spectrum of trace molecules, consequently reducing the signal-to-noise ratio of their fingerprint signals. Such a scenario poses a challenge for the accurate detection of trace molecules. To counteract the issue, the application of virtual gain provides an ideal and feasible solution. The synthesis of CFW has thus been identified as a promising approach to enhance the sensitivity in trace molecule sensing.

In a recently published paper in eLight, a collaborative team led by Prof. Shuang Zhang from the University of Hong Kong, Prof. Qing Dai from the National Center for Nanoscience and Technology, along with Prof. Na Liu from the University of Stuttgart, has unveiled a method for ultrahigh-sensitive molecular sensing^13^. The method is based on the application of synthesized complex-frequency excitation. The researchers constructed a complex frequency excitation from multiple real frequency responses with temporal truncated measurements. Here, the time truncation function is crucial in preventing energy divergence. Moreover, the sidebands resulting from time truncation are effectively eliminated through time averaging. The electric field ${E}_{T}({t}_{0})$ can be expanded as ${E}_{T}({t}_{0})=\frac{{E}_{0}}{2\pi }{\int }_{-\infty }^{+\infty }\frac{1}{i(\tilde{\omega }-\omega )}{e}^{-i\omega {t}_{0}}d\omega$. Naturally, the response in a quasi-steady state, under truncated CFW excitation, can be coherently synthesized from discrete real-frequency responses across a sufficiently broad spectral range. The final expression for the response under complex frequency excitation is denoted as $F(\tilde{\omega })\approx \sum _{n}F({\omega }_{n})\frac{1}{i(\tilde{\omega }-{\omega }_{n})}{e}^{i(\tilde{\omega }-{\omega }_{n}){t}_{0}}\varDelta \omega /2\pi$, where $F({\omega }_{n})$ represents the response at the real frequency. Note that both amplitude and phase information of $F({\omega }_{n})$ are essential. The phase component can be determined using the Kramers-Kronig relation for extraction^14^.

Figure 1 displays a comparative illustration of the current challenges and advancements in molecular sensing using graphene plasmons (GP)^15,16^. Figure 1a shows that while GP can enhance the interaction between light and molecules, the resulting signal in the extinction spectra of thin molecular layers remains notably weak. The phenomenon can be understood in terms of coupled harmonic oscillators^17^. Plasmon−phonon coupling generates two new hybrid modes, whose splitting distance depends on their coupling strength. At low concentrations, the intrinsic damping leads to a notably weak coupling strength between the plasmon and phonon, and the linewidth of the hybrid mode exceeds the splitting distance. It results in a substantial overlap between the two hybrid mode peaks, thereby obscuring subtle features in the extinction spectra. CFW can overcome the intrinsic damping loss, effectively restoring the molecular tiny responses. As demonstrated in Fig. 1b through numerical calculation, the application of synthesized CFW significantly amplifies the initially weak molecular vibrational response, showcasing a remarkable enhancement in detection sensitivity.

**Fig. 1:**
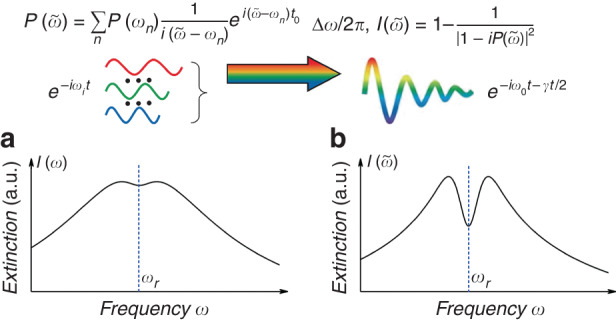
Illustration of damping compensation for sensing enhancement through synthesized CFW. **a** The extinction spectrum of the molecular layer enhanced by GP at real frequency. **b** The extinction spectrum of the molecular layer enhanced by GP at CFW

The work demonstrates the remarkable capability of synthesized CFW to significantly enhance molecular characteristic signals, thereby elevating the sensitivity ceiling of various sensors across diverse experimental contexts. This includes scenarios such as detecting deoxynivalenol molecules without plasmonic enhancement, as well as measuring silk protein molecules and bovine serum albumin protein solutions using graphene-based plasmonic sensors. The scalability and versatility of the synthesized CFW methodology hold immense promise for advancing the study of light-matter interactions. The breakthrough has the potential to unlock a wide array of applications in fields ranging from bio-detection and optical spectroscopy to biomedicine and pharmaceutical science, particularly within the realm of terahertz time-domain spectroscopy.
